# Research on the development of the concept of death in children and its influencing factors

**DOI:** 10.3389/fpsyg.2024.1376253

**Published:** 2024-05-10

**Authors:** Liu Jingjing, Liu Fuyan

**Affiliations:** ^1^Nanyang Institute of Technology, Central China Normal University, Wuhan, China; ^2^University of Chinese Academy of Sciences, Beijing, China

**Keywords:** mothers, concept of death, influencing factors, children, aged 5–6 years

## Abstract

In this study, 114 Chinese children aged 5–6 years old in Henan Province and 114 mothers were selected as research subjects, 59 boys, 55 girls, and 114 mothers. Semi-structured interviews were conducted with children to test the development of the concept of death in children. At the same time, a questionnaire survey was conducted among the children’s mothers to analyze the influencing factors of the development of children’s concept of death. The results of the study showed that children aged 5–6 years had the concept of death, and the sub-components were acquired in a certain order, but the development was not balanced, the overall level of development of the concept of death is slightly higher in girls than in boys; “Whether or not the family talked openly about the topic of death” had a significant effect on the sub-component of the children’s concept of death, “Universality of death” (*β* = −0.301, *t* = −2.007, *p* < 0.05) and “causality of death” (*β* = 0.315, *t* = 2.582, *p* < 0.05); and “whether or not there was the experience of exposure to death-related events” had a significant effect on “Irreversibility of death” (*β* = −0.326, *t* = −2.36, *p* < 0.05); Mothers’ natural acceptance of death (*β* = −0.293, *t* = −2.209, *p* < 0.05) and convergence-orientated acceptance (*β* = 0.415, *t* = 2.315, *p* < 0.05) had significant effects on the “inorganicity of death” of children’s concept of death.

## 1 Introduction

Goethe said, “As an essential ingredient of all the elements of life, death accompanies the whole of life, and any kind of death must be realized in a final act of life.” Death is a constitutive element of life, an inescapable issue, and a complex concept that encompasses cultural, emotional, religious, racial, and conceptual understandings (Slaughter, 2005). For children, do they know what death is? What do they think about death? American child education experts have long found that most children as young as 3 years old have already been exposed to the word “death”(段德智, 1991). Research into children’s concepts of death has been conducted since the 1920s, initially from a psychoanalytic perspective, and was mainly descriptive, using open-ended interviews, such as storytelling or drawing, to allow children to express themselves freely, to learn about children’s emotional reactions to death and to measure their understanding of death (Isaacs, 1929; Nagy, 1948; Von Hug-Hellmuth, 1965; Kane, 1977). In the 1960s and 1980s, children’s concepts of death were studied primarily from a cognitive perspective. Structured interviews were used with children to test their understanding and acquisition of the various sub-components of the concept of death. Based on his research, Kane (1979) classified the concept of child mortality into nine sub-components: realization, separation, immobility, irrevocability, causality, dysfunctionality, universality, insensitivity, and appearance (Kane, 1975). Hoffman and Strauss proposed five sub-components of the concept of death, universality, irreversibility, non-functionality, causality, and non-corporeal continuation (Schachter, 1992). Speece and Brent (1984), by analyzing more than 40 studies on the subject, grouped the concept of death into three sub-components: universality, irreversibility, and non-functionality (Speece and Brent, 1984). Such a research perspective and method of dividing the sub-components of the concept of death have also been used up to the present day (Guy, 1987; Orbach et al., 1987). The concept of death in children is characterized from the perspective of general cognitive development trend, trying to link the development of the concept of death with cognition (Safier, 1964; Koocher, 1973; White et al., 1978; Kane, 1979; Speece and Brent, 1984). Around the 1990s, new research content and methods emerged to study death as a biological concept (Carey et al., 1999). Emphasizing that the acquisition of domain-specific concepts may precede the overall development of cognitive levels, it is argued that the process of building children’s knowledge structures and the impact of domain-specific concepts on children’s cognitive development can be better understood through causal-explanatory models. For example, children at different ages were asked to make judgments about whether things that differed in some way (including humans, various animals, insects and inanimate things) had characteristics of life (e.g., being able to eat, breathe, experience emotions, reproduce, and die), and so on, pioneering the study of the development of children’s cognitive understanding of death from the perspective of the concept of biology. The factors influencing the development of children’s concept of death in this period have also attracted the attention of researchers, and a large number of studies have appeared on the relationship between children’s concept of death and variables such as age, gender, personal experience, peers, family socio-economic status, religious beliefs, and previous experience of death (Kane, 1979; Speece and Brent, 1984; Bull, 1985).

Previous research has produced several important findings and has advanced society’s understanding of and interest in the concept of a child and the factors that influence it (Kenyon, 2001). Children’s knowledge and understanding of death reduces their fear and anxiety of death (Slaughter and Griffiths, 2007), helps them to adjust to bereavement (Hoffman and Strauss, 1985), reduce suicide rates among children and adolescents (Orbach and Glaubman, 1978; Cuddy-Casey and Orvaschel, 1997), and can also prevent more deaths from occurring (McIntire et al., 1972). There is also a link between children’s grasp of the concept of death and children’s grasp of the concept of life, which can lead to a greater appreciation of life (Kastenbaum and Costa, 1977). Therefore, understanding children’s concepts of death and the factors that influence it has been a major concern for researchers and a key marker of progress in the social sciences and in the health field of psychology. Many researchers and practitioners have explicitly stated that death should be discussed with children in specific, explicit terms (Shapiro, 1994; O'Halloran and Altmaier, 1996; Luby, 2004). The study of the development of the concept of death in children and the factors influencing it will help to further the understanding and treatment of some profound issues of death, provide an empirical basis for death education, and shed some light on methods and approaches to death education, and the study will also have implications for clinicians or other practitioners who need to communicate with children about death.

## 2 Background

A review of the literature reveals that there are more studies on the concept of children’s death in the context of Western culture. In China, where the traditional culture is more profound, people regard death as a taboo, and there are fewer studies on death, and even fewer studies have been conducted on children, which further highlights the need for this study.

Most of the previous studies have been conducted with children in Western cultures and a broad sense (middle and upper primary schools, middle school, and high school students), while few studies have been conducted with 5–6-year-olds, and even fewer with Chinese children, who have a deeper traditional culture. Therefore, this study tested the development of the concept of death among 5–6-year-old Chinese children.

According to previous studies, the factors influencing the concept of death are broadly divided into two categories: individual influences factors (age, gender, personality, etc.) and socio-cultural influences factors (mass media, family religious beliefs, family socio-economic status, life experience, etc.). There is no research on the influence of “parental attitudes toward death” on the development of children’s concept of death. In China, mothers are the main caregivers for children, so this study will introduce the variable of mothers’ attitudes toward death to test the effects of children’s gender, exposure to death experience, religious beliefs, media, public talk about death, and mothers’ attitudes toward death on children’s conceptualization of death, to provide an empirical basis for children’s death education.

The following hypotheses are proposed in this study:

*H*1: Children at the age of 5–6 years already have the concept of death and with a certain sequence of acquisition.

*H*2: Variables such as children’s gender, exposure to death experience, religious beliefs, media, open discussion of death topics, and mothers’ attitudes toward death have a significant effect on the concept of death in 5–6-year-old children.

## 3 Materials and methods

### 3.1 Object of study

According to previous studies on death cognition, children have an understanding and awareness of death at the age of 3, and children at the age of 5–6 years old have reached a certain level of cognition of death; children at the age of 5–6 years old can understand language correctly and can express their thoughts clearly (McIntire et al., 1972; Panagiotaki et al., 2015). Therefore, in the present study, the research subjects were identified as 5–6 year old children and their mothers. The children aged 5–6 years were tested on the level of development of children’s concept of death, and their mothers were tested on the “Questionnaire on Factors Influencing the Development of the Concept of Death in Children.”

This study is part of a large-scale research project (Philosophy and Social Science Planning in Henan Province). The study selected Henan Province as the source of the sample and used accidental sampling to convene the testers in the large class of the public kindergarten L in city X in Henan Province, China. The kindergarten has five large classes, and each child has the opportunity to participate in the test. However, since children aged 5–6 years old are considered to be under-age, the informed consent of the children’s mothers was required before the survey. The mothers were given the “Semi-structured Interview Form for the Concept of Death of Children Aged 5–6 Years” and the “Questionnaire on Factors Influencing the Development of the Concept of Death of Children” by the classroom teachers and were informed of the purpose, methodology, significance, and precautions of the study, with the emphasis on the anonymity of the study, It was emphasized that the study followed the principles of anonymity, informed consent and voluntary participation. Inclusion criteria for study subjects: informed consent and voluntary participation. Exclusion criteria: more than 10% of missing items.

After the study was explained in detail to the mothers of the children in the older classes, the final number of children who agreed to participate was 123. Semi-structured interviews were conducted with the children who volunteered to participate in the test of the developmental level of children’s concept of death, and the “Questionnaire on Factors Influencing the Development of the Concept of Death of Children” was distributed to their mothers, and all the semi-structured interviews and questionnaires were completed within 2 weeks. There were 123 semi-structured interviews, 3 invalid semi-structured interviews were excluded, 120 valid semi-structured interviews were conducted, and the validity rate of the semi-structured interviews was 97.56%. 123 “Questionnaire on Factors Influencing the Development of the Concept of Death of Children” were retrieved, 6 invalid questionnaires were excluded, 117 valid questionnaires were retrieved, and the validity rate of the questionnaires was 95.12%. The validity of the “Semi-structured Interview Form for the Concept of Death of Children Aged 5–6 Years” in this study and the validity of the “Questionnaire on Factors Influencing the Development of the Concept of Death of Children” completed by the mothers were required to constitute the final valid study population. The final valid research subjects were 114 children aged 5–6 years and their mothers. The procedures followed were following the ethical and moral standards of the Sub-Committee on Human Research in Life Sciences of Central China Normal University. The composition of the study population in this study is shown in Tables 1, 2.

**Table 1:** List of basic information about the child.

Age	Boy	Girl	The total number of
5—6岁	59	55	114

**Table 2:** Summary of basic information about the child’s mother.

Basic information	Category	Number of people	Percentage (%)
Family role	Mother	114	100.0
Age level	Age 25 and under	0	0
	26–35 years old	72	63.2
	36-45 years old	38	33.3
	Age 46 and older	4	3.5
Degree of education	Technical secondary school (normal school, high school) and below	8	7.0
	Junior college	32	28.1
	Regular college course	56	49.1
	Master’s degree or above	18	15.8
Average monthly earnings	Less than 280 US dollars	7	6.1
	280–560 US dollars	38	33.3
	560–840 US dollars	50	43.9
	More than 840 US dollars	19	16.7
Number of children raised	One	49	43
	Two	59	51.8
	3 or more	6	5.2
Family structure	Single parent family	2	1.7
	Core family	46	40.4
	Stem family	62	54.4
	Joint family	4	3.5

1 US dollar ≈ 7.1873 RMB, 2024-03-13.

### 3.2 Research tool

This study adopts an empirical research paradigm, mainly using semi-structured interviews and questionnaires, and the research tools include the “Semi-structured interview form of the concept of death in children aged 5–6 years” and the “Questionnaire on Factors Influencing the Development of the Concept of Death of Children.”

#### 3.2.1 Semi-structured interview form for the concept of death of children aged 5–6 years

The “Semi-structured interview form of the concept of death in children aged 5–6 years” was designed by the researcher according to the needs of this study, based on the “Children’s Concept of Death Scale” developed by Taiwanese scholar Shu-Mei Zhang (張淑美, 2001), and previous studies (Lazar and Torney-Purta, 1991; Slaughter and Griffiths, 2007). consulting with experts and teachers, and designed independently according to the children’s linguistic habits and cognitive characteristics. The interview schedule categorized the concept of child death into five sub-components: Universality of death, Irreversibility of death, inorganicity of death, causality of death, and Determination of death, totaling 17 questions. The “Universality of death” is the belief that all organisms die; conversely, it is the belief that some organisms can avoid death, such as the belief that one’s self, one’s parents, or one’s best friend will not die. The “Irreversibility of death” means that it is believed that a person cannot be brought back to life and that death is permanent; conversely, it is believed that death is temporary, like sleep, and that it is possible to come back to life through certain methods. The “inorganicity of death” means that it is believed that when a person dies, the limbs and various organs of the body no longer have any function, such as not being able to walk, eat, sleep, etc. Conversely, it is believed that certain bodily functions are still present and that something can still be done. The “causality of death,” i.e., the belief that death is due to natural aging, disease, or external causes; conversely, the belief that death is a form of punishment or retribution. The “Determination of death” means being able to correctly judge death based on biological phenomena such as respiration, heartbeat, pulse, etc.; conversely, lying still or sleeping is regarded as death. Questions 1–5 test the universality of sub-component deaths, questions 6–10 test the irreversibility of sub-component deaths, and questions 11–15 test the inorganicity of sub-component deaths. Questions 16 and 17 are open-ended questions that test the causality of sub-component deaths and the determination of death (Slaughter and Griffiths, 2007).

In this study, the initial semi-structured interview schedule was first predicted for 30 children aged 5–6 years old, and the initial semi-structured interview schedule was statistically analyzed using SPSS 22.0 and AMOS 23.0 software, and the internal consistency reliabilities and split-half reliability coefficients of the five sub-components and the overall semi-structured interview schedule ranged from 0.741 to 0.902, and the sub-components correlation coefficients between them and the overall score are all between 0.709 and 0.917 and are significantly correlated at the 0.01 level, and the Cronbach’sαcoefficient is 0.956, which indicates that the semi-structured interview schedule has good reliability and validity. See Tables 3, 4 for details. The semi-structured interview scale was scored on a scale of 0–5 for each question, with higher scores indicating a more mature level of development of the concept of death, as shown in Table 5. One week after the completion of the pre-test, the class teacher and mothers of the respondents were followed up to confirm whether the children were experiencing any abnormal emotions and reactions. After confirming that there were no adverse reports, the formal administration of the test was begun to provide an overall picture of the children’s conceptual development of death.

**Table 3:** Load matrix of sub-components of semi-structured interviews and their indicators.

	Universality of death	Irreversibility of death	Inorganicity of death	Causality of death	Determination of death	Total
Number of sub-component items	5	5	5	1(open-ended question)	1(open-ended question)	17
Sub-component load range	0.489–0.785	0.519–0.816	0.697–0.776	0.612–0.745	0.521–0.767	–
Explanation rate (%)	49.598	5.767	4.121	3.768	3.532	66.786
α coefficient	0.778	0.912	0.902	0.895	0.919	0.956
α split-half reliability	0.741	0.902	0.811	0.895	0.901	0.892

**Table 4:** Matrix of correlation coefficients between the sub-components of the concept of child mortality and their correlation with the total score.

	Universality of death	Irreversibility of death	Inorganicity of death	Causality of death	Determination of death
Universality of death	0.612**	–	–	–	–
Irreversibility of death	0.529**	0.786**	–	–	–
Inorganicity of death	0.457**	0.523**	0.602**	–	–
Causality of death	0.520**	0.696**	0.981**	0.710**	–
Determination of death	0.709**	0.866**	0.889**	0.770**	0.917**

**P < 0.01.

**Table 5:** Scoring criteria of the semi-structured interview form on the concept of child death.

Sub-component	Question number	Standard or evaluation
Universality of death	1–5	Yes: 1; No: 0; Full maturity out of 5
Irreversibility of death	6–10	Yes: 1; No: 0;Full maturity out of 5
Inorganicity of death	11–15	Yes: 1; No: 0;Full maturity out of 5
Causality of death	16	Open questions: 1 point for a correct answer, up to 5 points
Determination of death	17	Open questions: 1 point for a correct answer, up to 5 points

Interviews with classroom teachers before interviewing children, knowing that children had recently watched the animated film 《The Lion King》, the interviews were introduced with the story of the animated film Lion King to keep children’s mood stable and relaxed. The detailed contents of the semi-structured interview form on the concept of child death are provided in Supplementary Appendix A.

#### 3.2.2 Questionnaire on factors influencing the development of the concept of death of children

This study developed its questionnaire using a top-down deductive research approach, i.e., reviewing a large number of previous relevant studies, developing the questionnaire dimensions, and designing the questionnaire items to form a formal questionnaire. The questionnaire consisted of two parts, the first part was basic information about the child, mainly including the child’s gender, exposure to death experience, media, whether the topic of death was talked about openly, and parents’ religious beliefs, totaling 9 items. The second part was the mother’s attitudes toward death, totaling 32 question items. A total of 32 items were included and the whole questionnaire was named “Questionnaire on Factors Influencing the Development of the Concept of Death of Children.”

Part I: Basic information about the child, with 9 questions, see Supplementary Appendix B for specific topics. Child’s gender: question 1; experience of exposure to the death event: questions 2, 3, and 4; media: questions 5, 7, and 8; whether or not the topic of death was talked about openly: question 6; and parents’ religious beliefs: question 9. Most of these questions were single choice and a few were multiple choice. This section is a qualitative variable, and to include it in the multiple regression analyses, this qualitative variable was transformed into a dummy variable and scored as shown in Table 6.

**Table 6:** Questionnaire on factors influencing the development of the concept of death of children the first part of the scoring criteria.

Content	Question number	Scoring formula
Gender	1	If girl, the value is 1 If boy, the value is 0
Have any experience with death-related events	2, 3, 4	If yes, the value is 1 If no, the value is 0
Have you been exposed to information about death through the media	5, 7, 8	If yes, the value is 1 If no, the value is 0
Whether the family talked openly about death	6	If yes, the value is 1 If no, the value is 0
Mother is whether there is a religion	9	If yes, the value is 1 If no, the value is 0

The part II: mothers’ attitudes toward death, the Death Attitude Profile Revised, DAP-R (DAP-R), revised by Gesser, Wong, and Reker in 1994 was chosen (Wong et al., 1994). Tang Lu Chineseised the Death Attitude Profile Revised, DAP-R to form a Chinese version in 2013 for assessing clinical nurses’ attitudes toward death (唐鲁, 2013). The scale has been widely used in China with different populations and is now a more comprehensive scale with higher reliability (Wong et al., 1994).The Death Attitude Profile Revised, DAP-R contains five dimensions of death fear (7 entries), death evasion (5 entries), natural acceptance (5 entries), convergence-oriented acceptance (10 entries), and escape-oriented acceptance (5 entries) with 32 entries in 5 dimensions, each of which was scored on a 5-point Likert scale: “Strongly Agree” = 5 points, “Agree” = 4 points, “Neutral “= 3 points, “Disagree” = 2 points, “Strongly Disagree” = 1 point, and the high or low score of the dimension indicates the high or low attitude toward the death of the tendency to that dimension, and the specific scoring criteria are as shown in Table 6. The Chinese version of the total table, the Cronbach’s alpha coefficient was 0.875, the folding coefficient was 0.864, and the Cronbach’s alpha coefficients of all dimensions were above 0.7. The scale has good reliability and validity (唐鲁, 2013; Table 7).

**Table 7:** Dimensions and grading criteria of the death attitude profile revised (DAP-R).

Dimensionality	Question number	Scoring formula
Fear of death	1, 2, 7, 18, 20, 21, 32	“Strongly agree” =5 points, “agree” =4 points, “neutral” =3 points, “disagree” =2 points, “strongly disagree” =1 point.
Death evasion	3, 10, 12, 19, 26	
Natural acceptance	6, 14, 17, 24, 30	
Convergence-oriented acceptance	4, 8, 13, 15, 16, 22, 25, 27, 28, 31	
Escape-oriented acceptance	5, 9, 11, 23, 29	

### 3.3 Research process

Firstly, the researchers in this study were professionally trained before the administration of the test to ensure that the test was conducted uniformly.

This study was approved by Central China Normal University, Ethic Committee, EC, Institutional Review Board (IRB Number 20221963). The entire study process was based on the ethical standards of clinical psychology research of “immediate research and timely intervention,” intervening in all aspects of the study to ensure that the subjects were not traumatized by the study itself. With some control and detection, no negative emotions were experienced by the subjects as a result of the study at the closing date.

Secondly, before the formal survey, the researcher first contacted the kindergarten director, explained the content of the survey in detail, and obtained the director’s consent, and then, with the assistance of the teachers in each class, explained the survey to the parents, obtained their informed consent, and, at the same time, explained in detail the way to fill out the questionnaire and the precautions to be taken by the parents. The “Semi-structured Interview Form for the Concept of Death of Children Aged 5–6 Years” was conducted by the researcher who led the children to a cozy activity room in the kindergarten, and the interview was conducted on a “one-to-one” basis, introduced by the animated cartoon “The Lion King” so that the children could easily respond to the content of the semi-structured interview form and record it. The interviewer’s questions were relatively structured, but the children’s responses were unstructured, and the researcher followed up on the children’s responses in the semi-structured interviews to find out the extent to which the children had a true understanding of the concept of death. The interviews lasted about 10 min or so, and the children’s emotional state was constantly monitored and prepared for reassurance during the interviews. The”Questionnaire on Factors Influencing the Development of the Concept of Death of Children” was released and collected by Questionstar, and the respondents were specified to be the mothers of the children, and the questionnaire was to be completed within 2 weeks. After the questionnaire was collected, invalid questionnaires with many missing values, short response times, and regular answers were strictly excluded according to the unified rules. Finally, based on data cleaning, the data results were statistically analyzed to verify the proposed hypotheses and construct a theoretical model.

### 3.4 Data processing

Score statistics and frequency counts of developmental maturity rate were conducted on the Structured Interview Schedule on Children’s Concept of Death. For the factors influencing the development of children’s concept of death, multiple stepwise regression analyses were conducted using SPSS 25.0 software.

## 4 Results

### 4.1 Development of the concept of death in children aged 5–6 years

#### 4.1.1 Development of the “universality of death” sub-component of the concept of death in children aged 5–6 years

The child mortality concept sub-component “Universality of death” has five test items, the highest score is 5, a score of 5 indicates that the sub-component is “fully mature,” a score below 5 indicates “immature,” a score of 0 indicates “does not have the sub-component concept,” and children who do not answer or say “do not want to say” are considered to avoid the question.

As can be seen from Table 8, children’s “universality of death” fully mature accounted for 55.3%, of which 55.2% were boys, girls 55.6%; immature accounted for 33.9%, of which 34.5% boys, girls 33.3%, which shows that the level of development of the universality of death is similar between boys and girls. Children who do not have the concept of this sub-component accounted for 5.4%, of which 6.9% were boys and 3.7% were girls; children who avoided the issue accounted for 5.4%, of which 3.4% were boys and 7.4% were girls; This means that the “do not have” rate is slightly higher for boys than for girls, while the “avoidance rate” is slightly higher for girls than for boys.

**Table 8:** Development of “universality of death” among children aged 5–6 years.

Universality of death	Total number of people (%)	Boy (%)	Girl (%)
Fully mature	55.3	55.2	55.6
Immature	33.9	34.5	33.3
Not available	5.4	6.9	3.7
Avoid	5.4	3.4	7.4
Total	100	100	100

According to the children’s responses during the interviews, most of the immature children with this sub-component believe that they will not die, citing the following reasons: “I’m still young!” or “I do not want to die yet!” This indicates that children of this age are aware that they will die in old age, but have an “avoidance” attitude toward their death.

#### 4.1.2 Development of the sub-component “irreversibility of death” in the concept of death in children aged 5–6 years

The concept of death sub-component “Irreversibility of death,” with five test items, was scored on the same scale as the sub-component “Universality of death.”

As shown in Table 9, nearly seven layers of children’s “irreversibility of death” reached full maturity, of which the rate of full maturity of boys is 69%, girls 66.7%; children’s immaturity rate of this sub-component is 30.4%, of which boys 31%, girls 29.6%; that is, the level of development of this sub-component is the same for boys and girls. Children do not have this sub-component accounted for 1.8%, of which boys 0%, girls 3.7%, girls slightly higher than boys; this sub-component of the test did not avoid the children.

**Table 9:** Development of “irreversibility of death” in children aged 5–6 years.

Irreversibility of death	Total number of people (%)	Boy (%)	Girl (%)
Fully mature	67.8	69.0	66.7
Immature	30.4	31.0	29.6
Not available	1.8	0	3.7
Avoid	0	0	0
Total	100	100	100

Among children with an immature sense of the “irreversibility of death,” the majority of boys responded to the question, “If a dead person is given a spell or incantation, will he or she come back to life?” to the question “Replied,” “Spells should be fine!”; Most girls, on the other hand, answered the question, “If you give a dead person some medicine, will he come back to life?” They answered, “Take some medicine!”

#### 4.1.3 Development of the sub-component of the concept of death in children aged 5–6 years “inorganicity of death”

The concept of death sub-component “inorganicity of death” is the same as the scoring criteria for the first two sub-components.

As can be seen from Table 10, children’s “Inorganic of death” fully mature accounted for 57.1%, of which 55.2% of boys, girls 59.2%, girls in this sub-component of the full maturity rate is slightly higher than that of boys; this sub-component of the immaturity of the children accounted for 42.9%, of which 44.8% boys, girls 40.8%; do not have the concept with the percentage of those who avoided it was 0%.

**Table 10:** Development of “inorganicity of death” in children aged 5–6 years.

Inorganicity of death	Total number of people (%)	Boy (%)	Girl (%)
Fully mature	57.1	55.2	59.2
Immature	42.9	44.8	40.8
Not available	0	0	0
Avoid	0	0	0
Total	100	100	100

Children, both boys, and girls, who are immature in the “inorganicity of death” answer the question, “Does a dead person still grieve?” The answer is “Yes!” The reasons for this are “Because he does not want to die!” “Because she will miss her father and mother!” etc. This is related to the developmental characteristics of children and corresponds to their “de-centeredness” and “empathy.”

#### 4.1.4 Development of the “causality of death” sub-component of the concept of death in children aged 5–6 years

The test questions for the concept of death sub-component “causality of death” are open-ended and are scored according to the number of correct answers given by the child, with one point awarded for each correct answer, up to a maximum of five points, with higher scores indicating a more mature development of the sub-component, whereas “do not know” is considered “do not have” and no answer is considered “avoidance.”

As shown in Table 11, 60.7% of children’s “Causality of death” has reached full maturity, of which 59.3% for boys and 62.1% for girls; the rate of immaturity of this sub-component is 26.9%, of which 25.9% for boys and 27.6% for girls. It means that more than half of the children’s concept of death sub-component “causality of death” has developed and matured, and the maturity rate of this sub-component is slightly higher in girls than in boys. The percentage of those who do not have this sub-component is 7.1%, of which 7.4% are boys and 6.9% are girls, and the percentage of those who avoid this question is 5.3%, of which 7.4% are boys and 3.4% are girls. The test question “causality of death” is open-ended, and children’s answers about the causality of death were diverse, as shown in Table 12.

**Table 11:** Development of “causality of death” in children aged 5–6 years.

Causality of death	Total number of people (%)	Boy (%)	Girl (%)
Fully mature	60.7	59.3	62.1
Immature	26.9	25.9	27.6
Not available	7.1	7.4	6.9
Avoid	5.3	7.4	3.4
Total	100	100	100

**Table 12:** Answers to “causality of death” in children aged 5–6.

Causality of death	Total number of people (%)	Boy (%)	Girl (%)	Children answer content examples
Aging	66.0	65.5	66.7	“People die when they are old” “They die when they live to be a hundred years old” “Hair is gray”
Illness	18.0	17.8	18.5	“Sickness died,” “Hand, foot, and mouth disease,” “Infectious disease,” “Too serious disease”
External attack	33.6	35.0	31.4	“Killed in a war,” “Suffocated,” “Killed,” “Killed,” “Shot to death”
Accidental disaster	23.2	22.2	25.9	“Earthquake crushed by house,” “Fall to death in dangerous places,” “Brick smashed to death, nails pierced”
Suicide	5.4	3.4	7.4	“Suicide” “Death by yourself”
Other	5.7	4.4	7.0	“Stay at home, cannot breathe fresh air,” “Starve,” “Die of thirst,” “Die of injections,” “Die if you do not exercise”
Do not know	5.4	3.4	7.4	“I do not know,” shaking his head
Avoidance	7.1	6.9	7.4	Do not speak

Some children say more than one reason.

According to the existing studies and the interview survey, the children’s answers to the question of “causality of death” are aging, illness, external attack, accident, suicide, other, do not know, and avoidance. As can be seen from Table 12, the most frequent answer of children on the causes of death is “aging,” accounting for 66.0%, followed by “external attack,” accounting for 33.6%, and then “accidental disaster,” accounting for 23.2%. The second is “external attack,” accounting for 33.6%, and the third is “accidental disaster,” accounting for 23.2%. Among the children’s answers, most of them can answer multiple causes, which shows that most of the children’s “causality of death” tends to mature development.

#### 4.1.5 Development of the sub-component “death determination” in the concept of death in children aged 5–6 years

The test question of the concept of death sub-component “Death determination” is also open, and the scoring standard is the same as that of “causality of death.”

As shown in Table 13, the full maturity rate of children’s “death determination” is 53.6%, of which 51.7% for boys and 55.6% for girls; the immaturity rate of this sub-component is 35.6%, of which 44.9% for boys and 25.9% for girls. The rate of maturation of this sub-component is slightly higher in girls than in boys. Among the five sub-components of the concept of death, “death determination” has the lowest maturity rate. The percentage of those who do not have this sub-component is 8.9%, of which 3.4% boys and 14.8% girls, and the percentage of those who avoid this question is 1.9%, of which 0% are boys and 3.7% are girls. Girls have more “not available” and “avoidance” than boys. The test question on “death determination” is open-ended, and children’s specific answers on the determination of death are shown in Table 14.

**Table 13:** Development of “death determination” in children aged 5–6 years.

Death determination	Total number of people (%)	Boy (%)	Girl (%)
Fully mature	53.6	51.7	55.6
Immature	35.6	44.9	25.9
Not available	8.9	3.4	14.8
Avoid	1.9	0	3.7
Total	100	100	100

**Table 14:** Answers to “death determination” of children aged 5–6.

Death verdict	Total number of people (%)	Boy (%)	Girl (%)
Cardiac arrest	1.8	3.4	0
Cannot breathe	5.4	10.3	0
Unable to move	60.7	62.1	59.3
Cannot listen/speak	44.6	48.3	40.7
Do not eat/drink	25.0	24.1	25.9
mistake	17.9	20.7	14.8
I do not know	8.9	8.9	14.8
Avoid	1.8	0	3.7

According to Table 14, children make their death decisions mostly based on the external physiological state of death, answering “not being able to move” the most often (60.7%), followed by not being able to hear or speak (44.6%). This is consistent with previous studies, which have shown that children between the ages of 5 and 10 are beginning to understand the biological facts about death (Lazar and Torney-Purta, 1991; Slaughter and Lyons, 2003; Poling and Evans, 2004).

### 4.2 Analysis of factors influencing the development of the concept of death in 5–6-year-old children

#### 4.2.1 Descriptive statistics

##### 4.2.1.1 Statistics on basic information on children

Basic information about the children was obtained through the first part of the “Questionnaire on Factors Influencing the Development of the Concept of Death of Children,” as shown in Table 15.

**Table 15:** Statistics on basic information on children.

	Experience of exposure to fatalities	Talk openly about death	Media	Parents’ religious beliefs
N	114	114	114	114
Yes	69(60.5%)	98(86%)	102(89.5%)	18(15.8%)
no	45(39.5%)	16(14%)	12(10.5%)	96(84.2%)

Tables 1, 15 show that of the 114 children, 59 (51.8%) were boys and 55 (48.2%) were girls. Sixty-nine (69), or 60.5%, had experience with death-related events; 45, or 39.5%, had no experience with death-related events. Ninety-eight (98) or 86% had talked openly about the topic of death; 16 or 14% had not talked openly about the topic of death. 102 (89.5%) were exposed to death-related information through the media; 12 (10.5%) were not exposed to death-related information through the media. Eighteen (15.8%) had a family religion and 96 (84.2%) had no family religion. The above were scored according to the scoring criteria in Part I of the “Questionnaire on Factors Influencing the Development of the Child’s Concept of Death” in Table 6, in preparation for the multiple stepwise regression analyses of the relationship between each of the influencing factors and each of the sub-components of the child’s concept of death.

##### 4.2.1.2 Basic information on mother’s attitude to death

Basic information on mothers’ attitudes toward death was obtained through the second part of the “Questionnaire on Factors Influencing the Development of the Concept of Death of Children.”

The descriptive statistics of mothers’ attitudes toward death were analyzed and the results are presented in Table 16. Among the 114 mothers in this survey, the mean values of the attitudes toward death scores ranged from 2.1407 to 3.8642. The escape-oriented acceptance dimension had the highest mean value of 3.8642 ± 0.64423 points. The natural acceptance dimension had the lowest mean value of 2.1407 ± 0.63201 Meanwhile the standard deviation of the scores of the dimensions ranged from 0.61871–0.64423 with a good degree of data dispersion. Mothers’ attitudes toward death were in the medium range according to their high and low ranges.

**Table 16:** Basic information on mother’s attitude toward death.

	Fear of death	Death evasion	Natural acceptance	Convergence-oriented acceptance	Escape-oriented acceptance
N	114	114	114	114	114
M	3.5000	3.1667	2.1407	3.7611	3.8642
SD	0.64423	0.61871	0.63201	0.61871	0.64423

SPSS25.0 software was used to conduct multiple stepwise regression analyses between the influencing factors and the sub-components of the concept of child death. The results were as follows:

#### 4.2.2 Multiple stepwise regression analyses of factors influencing the “universality of death”

As shown in Table 17, “talking openly about death” (yes, no) had a significant effect on children’s “Universality of death” (*β* = −0.301, *t* = −2.807, *p* < 0.05). The other factors did not have a significant effect on the sub-component “Universality of death.”

**Table 17:** Multiple stepwise regression analyses of factors influencing the “universality of death.”

	Gender	Experience of exposure to death-related events	Talk openly about death	Media	Parents’ religious beliefs	Attitude toward mother’s death				
						Fear of death	Death evasion	Nature acceptance	Convergence-oriented acceptance	Escape-oriented acceptance
Beta	−0.027	0.152	−0.301*	0.104	−0.095	−0.034	0.193	−0.214	−0.159	0.278
T	−0.179	1.007	−2.807*	0.669	−0.614	−0.150	0.901	−1.393	−0.846	1.557
F	4.008*									
R^2^_adj_	0.092									

Beta is the standardized regression coefficient, the determination coefficient adjusted R^2^_adj_. *p < 0.05.

#### 4.2.3 Multiple stepwise regression analyses of factors influencing “irreversibility of death”

According to Table 18, children’s experience of exposure to death-related events has a significant effect on “irreversibility of death” (*β* = −0.326, *t* = −2.36, *p* < 0.05). For example, children’s exposure to “death of a pet,” “death of a loved one,” and “attending a funeral” had a significant effect on “irreversibility of death.” significantly affect the “irreversibility of death.” The rest of the factors had no significant effect on the “irreversibility of death.”

**Table 18:** Multiple stepwise regression analyses of factors influencing “irreversibility of death.”

	Gender	Experience of exposure to death-related events	Talk openly about death	Media	Parents’ religious beliefs	Attitude toward mother’s death				
						Fear of death	Death evasion	Nature acceptance	Convergence-oriented acceptance	Escape-oriented acceptance
Beta	−0.014	−0.326*	0.121	0.212	0.187	0.133	−0.254	−0.013	0.268	−0.021
T	−0.089	−2.36*	0.775	1.311	1.152	0.568	−1.136	−0.083	1.364	−0.112
F	2.586*									
R^2^_adj_	−0.087									

Beta is the standardized regression coefficient, the determination coefficient adjusted R^2^_adj_. *p < 0.05.

#### 4.2.4 Multiple stepwise regression analyses of factors influencing “inorganicity of death”

According to Table 19, the natural acceptance dimension of mothers’ attitudes toward death (*β* = −0.293, *t* = −2.209, *p* < 0.05) and the convergence-oriented acceptance dimension (*β* = 0.415, *t* = 2.315, *p* < 0.05) had a significant effect on “inorganicity of death.” Other factors did not affect the “inorganicity of death.”

**Table 19:** Multiple stepwise regression analyses of factors influencing “inorganicity of death.”

	Gender	Experience of exposure to death-related events	Talk openly about death	Media	Parents’ religious beliefs	Attitude toward mother’s death				
						Fear of death	Death evasion	Nature acceptance	Convergence-oriented acceptance	Escape-oriented acceptance
Beta	0.020	−0.038	0.239	−0.043	0.158	−0.018	−0.112	−0.293*	0.415*	−0.167
T	0.141	−0.264	1.678	−0.290	1.069	−0.084	−0.551	−2.209*	2.315*	−0.980
F	3.557*									
R^2^_adj_	0.097									

Beta is the standardized regression coefficient, the determination coefficient adjusted R^2^_adj_. *p < 0.05.

#### 4.2.5 Multiple stepwise regression analyses of the factors influencing the “causality of death”

According to Table 20, “talking openly about death” has a significant effect on the sub-component “causality of death” (*β* = 0.315, *t* = 2.582, *p* < 0.05). Children are likely to be more likely to develop “causality of death” when there is open talk about death or death-related topics in the family. Other factors did not affect the “causality of death.”

**Table 20:** Multiple stepwise regression analyses of the factors influencing the “causality of death.”

	Gender	Experience of exposure to death-related events	Talk openly about death	Media	Parents’ religious beliefs	Attitude toward mother’s death				
						Fear of death	Death evasion	Nature acceptance	Convergence-oriented acceptance	Escape-oriented acceptance
Beta	−0.274	0.167	0.315*	−0.036	0.053	0.038	0.107	0.009	0.021	−0.205
T	−1.804	1.098	2.582*	0.231	0.335	0.165	0.494	0.057	0.111	−1.135
F	3.903*									
R^2^_adj_	−0.089									

Beta is the standardized regression coefficient, the determination coefficient adjusted R^2^_adj_. *p < 0.05.

#### 4.2.6 Multiple stepwise regression analyses of factors influencing the “determination of death”

According to Table 20, there is no significant effect of the influencing factors on the sub-component of the concept of death, “Determination of death.”

## 5 Discussion

The main purpose of this study was to understand the development of children’s concept of death and the order of acquisition of each sub-component through a cross-sectional study of children’s concept of death development, as well as the influence of factors such as children’s gender, experience with death-related events, religious beliefs, media, public talk about death, and mothers’ attitudes toward death on children’s concept of death development of the sub-components. The results showed that children aged 5–6 years had the concept of death, and the sub-components were acquired in a certain order, but the development of the sub-components was not balanced, which verified Hypothesis 1; children’s experience of death-related events, public talk about death, and the natural acceptance or tendency oriented acceptance dimensions of mothers’ attitudes toward death had a significant effect on children’s development of the concept of death, which verified Hypothesis 2. In this section, we will discuss the main findings in turn.

The concept of death is already present in children aged 5–6 years, and the sub-components are acquired in a certain order, but development is uneven. the overall level of development of the concept of death is slightly higher in girls than in boys.

Children aged 5–6 years have a concept of death and some have a fully developed concept of death. This is in line with Kane’s research (Kane, 1979), which found that 5-year-olds had death universality, death irreversibility, death inorganicity, and death causation (Kane, 1979). There is a certain order in the acquisition of the sub-components of the concept of death in 5–6-year-old children, and the present study found that the earliest sub-component to mature in children’s concept of death is the irreversibility of death, followed by the causality of death, and then by inorganicity of death, the universality of death, and judgment of death. This is consistent with study, in which children first understood the death conceptual sub-component “irreversibility of death” (Nagy, 1948; Walco, 1982; Lazar and Torney-Purta, 1991). This is inconsistent with research by Sigal Iron Hoffman and Sidney Strauss, who argued that the five sub-components of the concept of death are developed in the following order: universality, non-functionality, causality, irreversibility, and necessity (Hoffman and Strauss, 1985). The five sub-components of the concept of death in children aged 5–6 years are unevenly developed. According to the findings of this study, the development of the universality of death in children aged 5–6 years accounted for 55.3%, the development of irreversibility of death accounted for 67.8%, the development of inorganicity of death accounted for 57.1%, the development of the causality of death accounted for 60.7%, and the development of the determination of death accounted for 53.6%. The high rate of developmental maturity of death inorganicity and the low rate of developmental maturity of death determination indicate that the five sub-components are unevenly developed. This is consistent with previous studies that the five sub-components of the concept of death are not acquired at the same time, showing an unbalanced state (Speece and Brent, 1984; Kenyon, 2001; Slaughter, 2005). The overall level of development of the concept of death is slightly higher for girls than for boys. The levels of development of “universality of death” and “irreversibility of death” are more or less the same for both sexes, while the levels of development of “in organicity of death,” “causality of death” and “Death determination” are slightly higher for girls than for boys.

From the above findings, it is clear that death education for children is feasible, and that teachers, parents, clinicians, and psychologists should not underestimate the children’s tolerance. At the same time, when educating children about death, attention should be paid to the choice of content and gender differences, from easy to difficult, to adapt to the sequential and uneven characteristics of the development of the children’s concept of death in its various sub-components.

Children’s experience of exposure to death-related events, public talk about the topic of death, and natural or convergence-orientated acceptance of their mothers’ attitudes toward death had a significant effect on the development of children’s concepts of death.

Multiple stepwise regression analyses of the influences on each of the sub-components of children’s concept of death showed that children’s exposure to the experience of death had a significant effect on the development of “irreversibility of death.” This is inconsistent with the work of C. Randy Cotton and Lillian M. Range and Richard A. Jenkins and John C. Cavanaugh, who found that death experience was negatively related to the concept of death (Jenkins and Cavanaugh, 1986). Consistent with previous research by Hasazi, Bond, Kane, et al., children’s perceptions of death are influenced by the experience of death (Kane, 1979; Reilly et al., 1983; Jenkins and Cavanaugh, 1986; Bonoti et al., 2013). This variety of findings may be related in some way to the age of the children. Kane (1979) found that exposure to death appeared to affect only children under the age of 7 years, resulting in children exposed to death having a more mature view of death than those who were not. Children over the age of 7 years appeared to have reached a level of understanding of the concept of death, and therefore there was no effect (Matalon, 1998). Talking openly about death has a significant effect on children’s development of “death universality” and “death causality.” This is consistent with Matalon’s study, in which parents talking to children about death was significantly correlated with children’s understanding of death (May and Yalom, 1980). This shows that talking about the topic of death in the right way and with a good attitude toward family life is a good way to teach about death. Yalom believes that talking openly about the topic of death relieves children’s anxiety, fear, and worry about death, and can make them braver. Some studies have also shown that open family talks about death have a significant effect on children’s development of the “Universality of death” and “Irreversibility of death” of death, which may be related to the content of open family talk about death.

Natural and convergence-orientated acceptance of the mother’s attitude toward death had a significant effect on the sub-component of the child’s concept of death, “inorganicity of death.” Natural acceptance and convergence-orientated acceptance are positive attitudes, and the mother’s positive attitudes created a positive atmosphere for the children, which contributed to the development of the children’s concept of death. This is the most recent finding of the present study. It can be seen that teachers, parents, clinicians, psychologists, etc. should create a relaxed atmosphere when they need to talk to children about the topic of death; the content of death education should also be relevant to children’s lives, and the methods should be appropriate, such as children’s illustrated books, books, games, and art activities, which are important mediums and means of death education for children. According to the results of this study, it can be seen that mothers’ attitudes toward death affect children; therefore, parents should maintain positive attitudes toward death and skillfully seize incidental educational opportunities in their lives to promote the development of children’s concept of death.

According to Tables 17–21, the value of R^2^ is not very high because, in traditional Chinese culture, children tend to have positive connotations such as “seed,” “hope,” “growth,” etc. Adults seldom transmit the “concept” or “imagery” of death to preschoolers. and in the face of death, adults tend to tell children to avoid it or explain that they have gone to another, better world This is to allay children’s fear of death. However, this has resulted in preschoolers’ ignorance of death, leading to a relatively shallow knowledge and understanding of death. Therefore, in terms of the total model, these influences play a relatively small role in children’s perception of death, which is related to traditional Chinese culture. However, with the reform and development of education in China in recent years, life education has received the attention of the state and schools, and on 12 July 2021, the General Office of the Ministry of Education issued the Notice on Strengthening the Management of Students’ Mental Health, which explicitly states that schools should “pay attention to arranging a variety of forms of life education, frustration education, etc.,” and that “To effectively cultivate students’ psychological qualities of valuing life and loving life, and to enhance students’ sense of responsibility and mission.” Life education has increasingly become a hot research topic and trend, and people’s understanding of death will gradually deepen in favor of making young children aware of the whole process of life, so this study exploration in the context of Chinese culture to verify this trend and the existing development.

**Table 21:** Multiple stepwise regression analyses of factors influencing the “determination of death.”

	Gender	Experience of exposure to death-related events	Talk openly about death	Media	Parents’ religious beliefs	Attitude toward mother’s death				
						Fear of death	Death evasion	Nature acceptance	Convergence-oriented acceptance	Escape-oriented acceptance
Beta	−0.068	0.032	0.264	0.238	0.253	0.278	−0.072	−0.084	−0.063	0.127
T	−0.468	0.218	1.821	1.585	1.678	1.274	−0.349	−0.565	−0.344	0.736
F	1.357									
R^2^_adj_	0.064									

Beta is the standardized regression coefficient, the determination coefficient adjusted R^2^_adj_. *p < 0.05.

At the same time, our study also found that there were different levels of differences in children’s experience with death-related events, religious beliefs, the media, public discourse on death, and mothers’ attitudes toward death. Demographic variables such as the family’s economic level, mother’s education, family structure, and the number of children the family is raising can all influence these factors. The level of education, for example, has a direct impact on people’s knowledge and understanding of death and their attitudes toward it; the higher the level of education, the more open they are to death, and this behavior has a significant impact on their offspring, in a kind of continuity.

## 6 Conclusion

This study has made some new and striking findings. Firstly, Chinese children aged 5–6 years, who are deeply influenced by traditional Chinese culture, already possessed the concept of death, and the sub-components were acquired in a certain order, but the development of the sub-components was not balanced, and the overall development of the concept of death in girls was slightly higher than that in boys. Second, it was further verified that children’s experience with death-related events and public talk about death had a significant effect on the development of children’s concept of death; third, it was recently found that the natural acceptance or convergence-oriented acceptance of mothers’ attitudes toward death had a significant effect on children’s development of the concept of death.

## 7 Limitation

Then, this study also has many shortcomings. (1) in terms of the study population, the population of this study was only limited to 114 children aged 5–6 and their mothers in one region of Henan Province, and in future studies, we can involve more and wider regions, or even expand to the whole country, as well as make comparisons between different regions; (2) in this study, only mothers of 5-6-year-old children were investigated; therefore, we could not determine the effect of father’s attitudes toward death on children’s death conceptualization. Fathers may have different perspectives and roles compared to mothers, and thus the development of children’s concepts of death may differ according to differences in family roles. (3) Like other empirical studies, this study selected cross-sectional data at a certain point in time, whereas in reality the development of children’s concept of death is a long-term outcome influenced by various aspects, and longitudinal studies are needed to gain an in-depth understanding of the development of children’s concept of death and its influencing factors. (4) In addition, the development of children’s concept of death and the factors that influence it have not been studied in depth in this study, and there may be other variables (e.g., fathers’ attitudes toward death, intergenerational parenting, schooling, etc.), which will need to be continually explored in future studies.

## 8 Range of findings

Our study found that the development of the concept of child death and the factors influencing it have a positive significance for child death education in modern society with developed social media, surging social pressure, and rising youth suicide rates. Although death education has been widely carried out in the United States, Japan, and other countries. However, in countries such as China and India, death education has not been included in primary and secondary education so far, and even less in preschool education, believing that it will increase children’s fear of death, or believing that children are unable to understand the concept of death and that death education cannot be realized. However, through the results of foreign studies and the inference of this study, it can be found that children aged 5–6 years old already have the concept of death; this finding implies that it is feasible to provide children with death education, which will help schools and families to carry out reasonable and scientific death education according to their cognitive characteristics. In addition, children’s experience with death-related events, public talk about death, and natural or tendency-oriented acceptance of parents’ attitudes toward death have a significant impact on the development of children’s concept of death. This sheds light on the methods, approaches, and media used by parents, teachers, clinicians, and psychologists to implement death education. Rather than just reading from a book, we need to understand children’s psychology, feel their understanding of death, and grasp the factors that influence the development of children’s concept of death to successfully promote the implementation of death education. Of course, the application of these findings is not limited to the Chinese cultural context, and these preliminary but important findings can be tested in other socio-cultural contexts as well.

## Data availability statement

The data analyzed in this study is subject to the following licenses/restrictions: data will be made available on request. Requests to access these datasets should be directed to 431164358@qq.com.

## Ethics statement

This study was approved by the Central China Normal University, Ethic Committee, EC, Institutional Review Board (IRB Number 20221963). Written informed consent was provided by the participants legal guardian/next of kin.

## Author contributions

LF: Writing – review & editing. LJ: Writing – original draft.
