# Supplementary material for: Research on the development of the concept of death in children and its influencing factors
Source: Front Psychol. 2024 May 10;15:1376253. doi: 10.3389/fpsyg.2024.1376253 (PMC11122897; doi:10.3389/fpsyg.2024.1376253)
Supplement: Supplementary file 1 [file Data_Sheet_1.docx]

**Appendix A**

Semi-structured interview form of the concept of death in children aged 5-6 years

In the cartoon The Lion King, Simba's father is full of love for Simba and guides him to be more and more brave, but where does Simba's father go in the end (Simba's father dies in the cartoon)? Through the exchange of cartoons, the topic of the interview is naturally introduced.

1. Is everyone going to die? Yes No
2. Will I （tester）die someday? Yes No
3. Will the children in your class die one day? Yes No
4. Will your father and mother die one day? Yes No
5. Will you die one day? Yes No
6. When a man dies, can he come back to life? Yes No
7. If a man dies and you give him water, will he come back to life? Yes No
8. If a man dies and you give him something to eat, will he come back to life? Yes No
9. If a man dies and you give him medicine, will he come back to life? Yes No
10. A man dies, with spells, will he come back to life? Yes No
11. If a man dies, will he do the same things as he did when he was alive？ Yes No
12. If a man dies, does he still move? Yes No
13. If a man is dead, can he still talk? Yes No
14. If a man is dead, does he still hear? Yes No
15. If a man dies, will he still be sad? Yes No
16. Why do people die? (Open question, can answer multiple answers)
17. How do you tell someone that they're dead? (Open question, can answer multiple answers)

**Appendix B**

The specific items in the first part are as follows:

1. What is the sex of your child? A boy B-girl
2. Has your child experienced the death of his or her favorite pet? A has it B doesn't
3. Has your child experienced the death of someone around them? A has it B doesn't
4. Have your children ever attended a funeral? A Yes B No
5. In what ways is your child usually exposed to experiences related to death? (Multiple options available)

A When a pet dies B when a relative dies C when watching media reports

D when watching cartoons or TV episodes E when reading picture books

F video or online games

1. Have you ever talked about death with your children?

A took the initiative to talk with the child B Talked about it when the child asked

C didn't talk about it

7. When have you talked about death with your children? (Multiple options available)

A When a pet dies B when a relative dies C when watching media reports

D when watching cartoons or TV episodes E when reading picture books

F video or online games G small talk H other I did not talk about

8. Through what media do you explain death to your children?

A children's picture book B Television media C language statement

D example E perfunctory

9. What is your religion?

A Buddhist B Christian C Catholic D Taoist E Islamic F Other G None
